# Regulatory T cells inhibit Fas ligand-induced innate and adaptive tumour immunity

**DOI:** 10.1002/eji.200636593

**Published:** 2007-03

**Authors:** Anna Katharina Simon, Emma Jones, Hannah Richards, Kate Wright, Gareth Betts, Andrew Godkin, Gavin Screaton, Awen Gallimore

**Affiliations:** 1 Medical Biochemistry and Immunology, School of Medicine, Cardiff UniversityCardiff, UK; 2 Nuffield Department of Medicine, John Radcliffe HospitalOxford, UK; 3 Hammersmith Hospital, Imperial CollegeLondon, UK

**Keywords:** Fas ligand, Innate tumour immunity, Regulatory T cells

## Abstract

CD4^+^CD25^+^ regulatory T cells (Treg) are known to influence T cell responses to tumours. Here we have explored the role of Treg in inhibiting not only adaptive, but also innate immune responses to tumours. To this end we used a Fas ligand (FasL)-expressing melanoma cell line in a mouse model. In this system, innate immunity is sufficient to reject the tumour. All mice depleted of Treg and challenged with FasL-expressing melanoma remained tumour-free. Investigation of the underlying cellular effector mechanisms revealed that depletion of Treg enhanced an NK cell response capable of tumour lysis. Furthermore, this initial innate immune response primed mice to make an effective adaptive immune response leading to complete rejection of challenge with the parental melanoma. Both antigen-specific antibody and CD4^+^ T cells were implicated in protection *via* adaptive immunity. We conclude that removal of Treg and vaccination with whole tumour cells expressing FasL activates multiple arms of the immune system, leading to efficient tumour rejection. These findings highlight a novel role for FasL in inducing innate immune responses that are normally inhibited by Treg and uncover an adjuvant effect of FasL that can be used to stimulate tumour immunity after depletion of Treg.

## Introduction

Many studies in mouse models indicate that CD4^+^CD25^+^regulatory T cells (Treg) significantly impact the development of anti-tumour immune responses [1]. Moreover, Treg exhibiting suppressive activity have been found within tumour-infiltrating lymphocytes of cancer patients [2]. Accumulation of CD25^+^ regulatory cells within tumours correlates with reduced survival of patients with ovarian cancer, arguing for a critical role for Treg in disease progression [3]. Treg identified in humans exhibit the same phenotypic and functional characteristics as their murine counterparts. These cells express the transcription factor FoxP3 as well as high levels of CTLA-4 and suppress the proliferation of conventional T cells *in vitro*[4]. Clinical trials are underway to test whether depletion of Treg can improve immunity to ovarian cancer [5].

Here we use a model of tumour rejection involving a melanoma cell line expressing Fas ligand (B16FasL) to study the effect of Treg on both innate and adaptive immune responses. In a previous study, we found that overexpression of FasL in the melanoma cell line B16 leads to enhanced rejection [6]. Similar results have been obtained with other tumour cell lines genetically manipulated to over express FasL, and rejection is associated with the induction of marked inflammation with extensive neutrophil infiltration [7]. Recent studies have elaborated upon this result and show that in this system membrane-bound FasL mediates inflammation [8], CXC chemokines are essential for the observed neutrophil recruitment [9], and neutrophil apoptosis mediated *via* FasL expression is crucial for the induction of inflammation [10]. Of additional relevance, we demonstrated previously that B16FasL-treated mice that remained tumour free were able to reject a secondary challenge with the parental tumour B16F10 *via* induction of an antibody response [6]. B16FasL is therefore a well-characterised model of tumour immunity, involving an initial innate immune response followed by the establishment of an adaptive immune response capable of rejecting a secondary challenge, and is therefore ideal for the study of the inhibitory effect of Treg on tumour immunity.

Although the above-cited studies provide an in-depth characterization of the B16FasL model, none directly addressed the cellular effector mechanisms by which the tumour itself is eliminated. Here, we show that the innate immune response is sufficient to mediate the rejection of B16FasL by recruitment of NK cells capable of tumour lysis. Furthermore, we show that depletion of Treg enhances adaptive immune responses by uncovering a previously undetectable CD4^+^ T cell response. The information obtained will be useful in the design of vaccine strategies aimed at inducing multiple arms of the immune system and for monitoring anti-tumour responses in clinical trials of Treg depletion.

## Results

### Innate immune responses are critical for rejection of FasL-expressing melanoma

**Figure 1:**
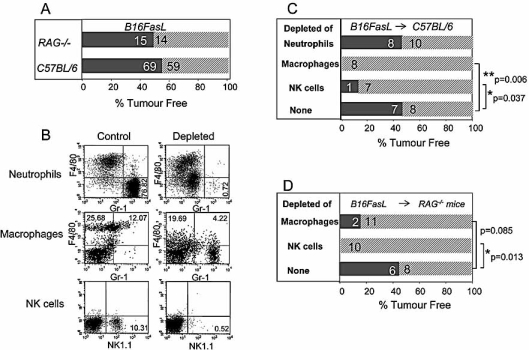
The innate immune system plays a key role in rejection of FasL-expressing tumour cells. (A, C, D) Mice were inoculated s.c. with B16FasL, and tumour growth was measured for a minimum of 50 days. Solid bars and hatched bars indicate numbers of tumour-free mice and tumour-bearing mice, respectively, in (A) B6 and RAG^–/–^ mice, (C) B6 mice depleted of neutrophils, macrophages or NK cells or left untreated (none) and (D) RAG^–/–^ mice depleted of macrophages or NK cells or left untreated (none). Each experiment was performed on two separate occasions. The statistical analysis was performed using Fisher's exact test contingency tables with Prism software. (B) Depletion of neutrophils (F4/80^–^Gr-1^hi^), macrophages (F4/80^hi^) and NK cells (NK1.1^+^) in B6 mice treated with RB6–8C5, Carrageenan or PK136, respectively, compared to control GL113-treated mice was evaluated by FACS. FACS plots shown are representative of ten mice per group.

Several studies, including our own previous work, have shown that injection of mice with FasL-expressing tumour cells induces an inflammatory response within hours of injection [11–13]. In order to determine whether innate immunity is sufficient for the rejection of B16FasL, the cells were injected into both C57BL/6 (B6) and C57BL/6RAG ^–/–^ (RAG^–/–^) mice and tumour growth monitored. Approximately 50% of mice in each group rejected the tumour challenge, suggesting that neither T nor B lymphocytes played an important role in the rejection of B16FasL and that the innate immune response was sufficient (Fig. 1A). We then determined which type of innate immune cells was responsible for this rejection by depleting neutrophils, macrophages and NK cells *in vivo* prior to tumour inoculation (Fig. 1B). Macrophages and NK cells but not neutrophils played a critical role in the rejection of B16FasL in B6 mice, as their depletion significantly inhibited rejection (Fig. 1C). However, in RAG^–/–^ mice only depletion of NK cells significantly affected rejection, whereas depletion of macrophages reduced the number of tumour-free mice substantially but not to a statistically significant level (Fig. 1D). These data highlight a role for innate immune cells in B16FasL rejection and indicate that NK cells do not have to interact with T cells in order to mediate tumour rejection. However, to formally demonstrate a role for these cells, we wished to carry out functional assays. Since isolation of sufficient viable cells from the subcutaneous tumour site is difficult, we established an *in vivo* peritoneal challenge model similar to the one described by Hohlbaum *et al.* [8.].

To identify the effector cells responsible for tumour killing, B16FasL, control B16F10 cells or PBS were injected into the peritoneum. The lavage was subsequently evaluated for its cellular composition and capacity to lyse tumour cells *in vitro*. Both B16F10 and B16FasL, but not PBS, attracted macrophages (20–25% of total cells in lavage) and NK cells (7–10%) (data not shown). Strikingly, and as published by Hohlbaum *et al.*, only B16FasL attracted a large number of neutrophils (50%) into the peritoneal cavity as compared to 10% in B16F10-or PBS-treated mice [13]. These numbers were similar in B6 and RAG^–/–^ mice (data not shown).

**Figure 2:**
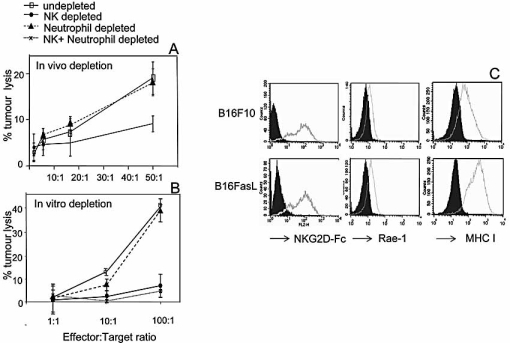
NK cells recovered from the peritoneum of mice injected with B16FasL kill tumour cells *in vitro*. Peritoneal cells were recovered 18 h after i.p. injection of B16FasL and used as effectors in a ^51^Cr-release assay against B16F10 target cells (similar results were obtained using B16FasL as target cells, data not shown) at different effector to target ratios. Data shown are the mean ± SD of five mice per group. (A) *In vivo* depletion of neutrophils or NK cells. (B) Prior to the killing assay, effectors were depleted*in vitro* of neutrophils or NK cells or both. (C) B16F10 and B16FasL were immunostained for MHC class 1, Rae-1 and NKG2D ligands (open histograms), evaluated by FACS and compared to recombinant protein/isotype controls (filled histograms). These stainings were performed twice with similar results.

Cells obtained from the lavage of PBS-treated mice did not efficiently lyse tumour cells *ex vivo* (data not shown). However, cells isolated from B16FasL-challenged peritoneal lavage were able to lyse B16F10 and B16FasL. We hypothesized that either NK cells or neutrophils were eliminating the tumour cells. To investigate this we depleted B6 mice *in vivo* with the mAb PK136 that depletes NK1.1^+^ cells or with the neutrophil-depleting mAb RB6–8C5.*In vivo* depletion of NK cells but not neutrophils decreased the *ex vivo* lysis of the tumour by the peritoneal cells (Fig. 2A). Similarly, depletion of NK cells but not of neutrophils *ex vivo*from the peritoneal exudate completely abolished this killing (Fig. 2B), also excluding the possibility that macrophages are a major player in tumour lysis. Since NK cells are known to recognise both the absence of MHC class I and an additional stimulatory signal through the NKG2D receptor, we tested whether our tumour cell lines express NKG2D ligands using an NKG2D-Fc fusion protein or GITR-L-Fc followed by a fluorescently tagged anti-human Ig Ab. Both B16F10 and B16FasL stained positively with recombinant NKG2D-Fc and were positive for the murine NKG2D ligand Rae-1 (Fig. 2C). The tumour cells also expressed low levels of MHC class I compared to spleen cells (Fig. 2C and data not shown). We therefore concluded that NK cells are recruited upon B16FasL inoculation and are the key effector cells responsible for tumour lysis.

### CD4^+^CD25^+^ regulatory cells inhibit tumour-induced innate immune responses

Our hypothesis that Treg inhibit innate immune responses was first formulated on the basis of an observation made using parental B16F10 cells. In these experiments RAG^–/–^ mice were injected with CD4^+^CD25^+^ Treg cells or control CD4^+^CD25^–^ cells, both from naive mice, or PBS alone, followed by B16F10 tumour challenge. Although all mice grew tumours, we observed that tumours grew more rapidly in mice that had received Treg compared to mice that received the control cell population or PBS (data not shown). Since the results of this experiment strongly suggested that Treg inhibit innate immune responses, we decided to perform proof of principle experiments using B16FasL to address this point. B16FasL were selected for the following reasons: i) B16FasL, as opposed to B16F10, is rejected in RAG^–/–^ mice as efficiently as in B6 mice; ii) the innate immune response to B16FasL is sufficient to reject the tumour (Fig. 1); iii) we have identified NK cells as the main effector cell type mediating this innate B16FasL immunity in both B6 and RAG^–/–^ mice.

**Figure 3:**
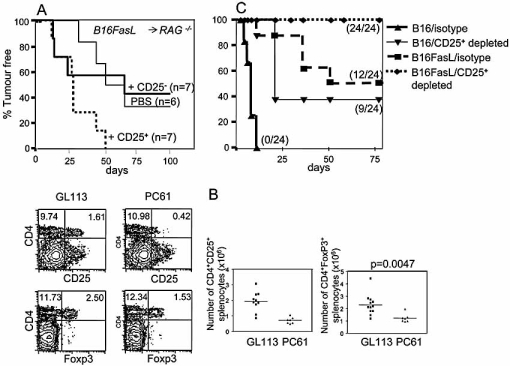
CD4^+^CD25^+^ regulatory cells inhibit the innate rejection of B16FasL. (A) RAG^–/–^ mice were injected with either PBS (thin solid line), 1 × 10^6^ purified CD4^+^CD25^–^ T cells (thick solid line) or 1 × 10^6^ purified CD4^+^CD25^+^ T cells (short dashes) 1 day prior to s.c. injection of B16FasL cells. Tumour growth was monitored over time. The number in parenthesis indicates the number of mice per group. Similar results were obtained on two separate occasions with a minimum of six mice per group. (B) B6 mice were injected i.p. with control Ab GL113 or anti-CD25 depleting Ab PC61. Spleens were harvested 3 days later and immunostained for CD4, CD25 and FoxP3 and evaluated by FACS. Plots shown are representative of ten mice per group. Total numbers of CD4^+^FoxP3^+^ and CD4^+^CD25^+^ cells in the spleen are also given with a minimum of seven mice per group. (C) B6 mice were either depleted of CD25^+^ cells or not (GL113 isotype control) and then injected s.c. with parental B16F10 or B16FasL. Tumour growth was monitored over 75 days. Tumour-free mice are indicated. This experiment was repeated five times with a minimum of eight mice per group. The number of tumour-free mice in the experiment shown is indicated in parenthesis.

We investigated this hypothesis by injecting RAG^–/–^ mice with CD4^+^CD25^+^ or CD4^+^CD25^–^ cells purified from B6 mice, followed 1 day later by inoculation with B16FasL. Tumours were monitored weekly for at least 100 days in all mouse groups. Approximately 50% of the mice inoculated with CD4^+^CD25^–^ cells or PBS rejected the B16FasL inoculum. However, rejection was not observed in any of the mice injected with CD4^+^CD25^+^ cells. Transfer of CD4^+^CD25^–^ T cells had no effect on tumour development (Fig. 3A). These data suggest that CD4^+^CD25^+^ Treg inhibit elements of the innate immune system, reducing their ability to mediate tumour rejection.

To further characterise this effect, we investigated whether tumour rejection can be improved by depleting Treg in B6 mice with the CD25-specific mAb PC61. Administration of 1 mg PC61 results in an approximate 4-fold reduction in the number of CD4^+^CD25^+^cells and an approximate 2-fold reduction in FoxP3^+^ T cells for around 3 wks after injection (Fig. 3B). B16FasL tumours are rejected in 50% of untreated mice, increasing to 100% once Treg are depleted. By contrast, no B16F10 melanoma rejection is observed unless Treg are depleted, after which rejection rates are enhanced significantly (Fig. 3C). Collectively these data indicate that CD4^+^CD25^+^ Treg inhibit innate immune responses that are capable of tumour rejection in both B6 and RAG^–/–^ mice. Since NK cells were the only cell type shown to be critical for tumour rejection in both B6 and RAG^–/–^ mice, we postulated that Treg cells inhibit the activity of B16FasL-induced NK cells.

### Treg inhibit NK cell activity

**Figure 4:**
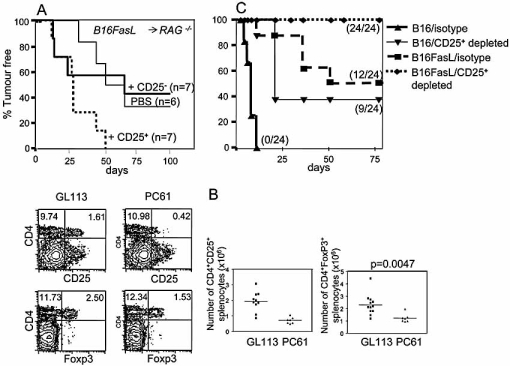
CD4^+^CD25^+^ cells inhibit killing and attraction of NK cells in the peritoneal model. (A) B16FasL cells (2 × 10^6^) were injected into B6 mice, and 24 h later the peritoneum was lavaged and cells counted. Lavaged cells (10^5^) were added to 10^4^ ^51^Cr-labelled B16F10 cells in the presence of 10^5^ purified CD4^+^CD25^+^ or CD4^+^CD25^–^ T cells. Controls included lavaged cells (effectors) alone and CD4^+^CD25^–^ cells without effectors. The supernatant was collected 5 h later and analysed on a beta counter. Means were calculated from triplicates. (B) CD4^+^CD25*^+^*or CD4^+^CD25^–^ cells (2 × 10^6^) were injected i.p. concomitantly with 2 × 10^6^ B16FasL. Peritoneal cells were recovered 18 h later and used as effectors in a killing assay (as above) against B16F10 at different effector to target ratios. This was repeated in a second experiment (three mice per group in each experiment). (C) B6 mice were depleted *in vivo* of CD25^+^ cells, NK cells or both CD25^+^ cells and NK cells. Treatment with the Ab GL113 served as a control (no depletion). The mice were rested until the depleting Ab PC61 had disappeared from the serum and were then injected i.p. with B16FasL cells. Peritoneal cells were used as effectors cells as in (A). (D) NK1.1^+^CD3^–^ cells were evaluated by FACS. Shown are two separate experiments: In the left panel, recipient mice were RAG^–/–^ (*n*=2 per group), and in the right panel, recipient mice were B6. Data are representative of two separate experiments with a minimum of five mice per group. Statistical analysis was performed using the two-tailed Mann-Whitney test with Prism software (**p<0.01, *p<0.05).

To determine whether Treg inhibit tumour lysis by NK cells, peritoneal cells were obtained from B16FasL-injected mice. CD4^+^CD25^+^ cells or CD4^+^CD25^–^ cells purified from naive B6 mice were either left unstimulated and added 4 h before the killing assay to the peritoneal lavage or were stimulated with anti-CD3 Ab and irradiated APC. Although another study showed that Treg can inhibit NK killing of tumours using unstimulated CD4^+^CD25^+^ cells [14], we only observed inhibition of NK cell killing when the CD4^+^CD25^+^ cells were stimulated with anti-CD3 Ab and irradiated APC (Fig. 4A). Similar findings were reported by Smyth *et al*. [15]. These cells also inhibited proliferation of CD4^+^CD25^–^ cells in a conventional Treg suppression assay (see *Materials and methods*). To confirm this finding *in vivo*, we purified CD4^+^CD25^+^ cells or CD4^+^CD25^–^ cells and injected them concomitantly with B16FasL tumour cells into the peritoneum of B6 mice. The presence of Treg *in vivo* significantly inhibited *ex vivo* lysis by the peritoneal exudate cells (Fig. 4B). Conversely, when Treg were depleted *in vivo*, tumour lysis *ex vivo* was increased (Fig. 4C). Taken together, these data show that Treg suppress NK cell tumour lysis *in vivo* and *in vitro* after stimulation with anti-CD3 Ab and irradiated APC. In addition to the suppressive effects of the Treg on killing, we observed an approximate 2-fold reduction of NK cell percentages in the peritoneal lavage when RAG^–/–^ mice (Fig. 4D, left panel) or B6 mice (Fig. 4D, right panel) were injected with CD4^+^CD25^+^ as opposed to CD4^+^CD25^–^ cells. Altogether, these results suggest that in addition to a direct effect of Treg on the killing capacity of NK cells, Treg also either inhibit recruitment of NK cells to sites of inflammation or promote death of NK cells.

### Enhanced adaptive immunity in the absence of CD25^+^ regulatory cells

**Figure 5:**
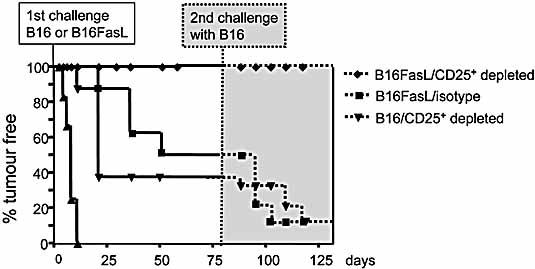
The adaptive immune response to B16FasL is inhibited by CD25^+^ cells. Mice that remained tumour-free in Fig. 3B were challenged s.c. with 2 × 10^5^ B16F10 in the same flank, and tumour growth was measured for another 50 days. This experiment was repeated five times with a minimum of eight mice per group.

**Figure 6:**
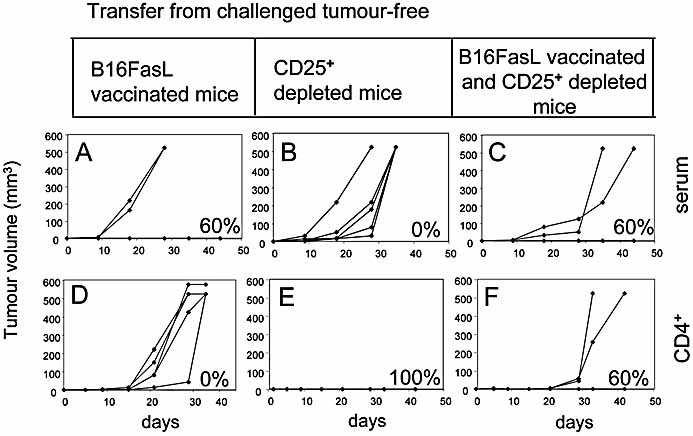
Both antibody and CD4^+^ cells mediate B16FasL tumour immunity in CD4^+^CD25^+^-depleted mice.Serum (A–C) or CD4^+^ cells (D–F) were transferred from mice vaccinated with B16FasL, depleted of CD25^+^ cells or both into naive B6 mice, which were subsequently challenged with B16F10. Each line represents tumour growth in an individual mouse, and the numbers in the lower right corner indicate the percentage of tumour-free mice in each group. Similar results were obtained in five independent experiments with a minimum of five mice per group.

Having established that the innate immune response to B16FasL was improved following removal of Treg, we next sought to determine whether this might have an effect on the adaptive immune response. To this end we challenged mice vaccinated with either B16FasL or B16F10 cells (from Fig. 3C) with the parental cell line B16F10, which is not normally rejected. About 50% of mice that received B16FasL or B16F10 and anti-CD25 rejected the B16F10 cells. Strikingly, all mice that received B16FasL and anti-CD25 rejected the B16F10 challenge (Fig. 5, 2^nd^ challenge), implying an additive or synergistic effect between Treg depletion and adaptive immunity elicited by tumour vaccination. This is in accord with our previous observation that the secondary response to the parental B16F10 tumour in mice injected with B16FasL is due to a T helper-dependent Ab response [6]: following depletion of Treg**,** inoculation of mice with B16F10 cells results in stimulation of CD4^+^ T cell responses capable of tumour rejection in approximately 70% of vaccinated mice [16]. On the basis of these results, we postulated that the improved rejection in mice with vaccination combined with Treg depletion was due to an improved Ab response, improved T cell response or a complementary action of both arms of the immune system. To identify the correlates of immunity, serum, CD4^+^ T cells or CD8^+^ T cells obtained from vaccinated mice were transferred into naive hosts, which were then challenged with the parental cell line B16F10. Tumours grew in all mice that received no serum or cells and in all mice receiving CD8^+^ T cells purified from the vaccinated mice (data not shown). Fig. 5 shows that serum from B16FasL-vaccinated mice but not naive mice, as observed previously [6], mediated rejection of the parental tumour B16F10 in a proportion of the naive recipient mice: 60% of mice remained tumour-free (Fig. 6A), whereas upon adoptive transfer, CD4^+^ T cells purified from the same mice could not protect from a B16F10 challenge (Fig. 6D). Furthermore, serum from unprimed mice depleted of Treg was not protective (Fig. 6B). However, all mice remained tumour-free when CD4^+^ T cells were transferred (Fig. 6E), as shown previously [16]. Finally, both CD4^+^ T cells and serum from mice vaccinated with B16FasL in the absence of Treg promoted 60% of mice to reject B16F10 (Fig. 6C, F). Collectively, these data indicate that tumour rejection was more efficient in mice injected with B16FasL following depletion of Treg due to stimulation of both a protective Ab and a CD4^+^ T cell response.

## Discussion

It is emerging that one of the most promising human applications of Treg is in tumour immunology, in large part because depletion of Treg should prove easier than *ex vivo* expansion and cell transfer for the treatment of autoimmune disease. It is therefore extremely important to understand the implications of Treg depletion on both the innate and adaptive responses to tumours. FasL-transfected murine melanoma is ideally suited to study the effect of Treg depletion on innate immune responses, as it (in contrast to the untransfected tumour cell line) induces an influx of inflammatory cells, which leads to rejection of the FasL melanoma in the absence of adaptive immunity [8, 17]. We show here that the innate immune system is sufficient to reject B16FasL tumours, since tumour cells were rejected as efficiently in RAG**^–/–^** mice as in wild-type B6 mice. Furthermore, rejection of B16FasL in B6 mice requires NK cells and macrophages. Macrophages attracted to the site of B16FasL inoculation may promote tumour rejection directly through production of inflammatory cytokines and recruitment of other immune cells. Indeed, engagement of Fas^+^ macrophages with FasL has been shown to induce secretion of CC and CXC chemokines, which act as chemoattractants for NK cells that clearly play an important role in rejection of B16FasL [9]. It is also possible that the cells, as shown by others, secrete chemokines that attract Treg to the site of tumour, possibly promoting their death through interaction with FasL on the tumour cells. NK cells most likely promote tumour rejection through direct cytolysis. In the peritoneal model, recruited NK cells but not neutrophils lysed the parental and FasL-transfected melanomas. This is in contrast to a publication by Seino *et al.* in which neutrophils from peritoneal exudates elicited by injection of FasL-expressing lymphoma were suspected to be directly cytotoxic against tumour cells [7]; however, this was not directly demonstrated but rather implied on the basis that the exudate contained 80 to 90% neutrophils [7]. Our findings are consistent with a report by Igney *et al*. in which mice with a deficiency in neutrophil cytotoxicity were injected with FasL-expressing tumours, and the tumour growth rate was the same as in wild-type mice [18].

One explanation for the ability of FasL expression to help tumour rejection is preferential killing of Treg. We performed a killing experiment using B16FasL or recombinant crosslinked FasL, which indicated that CD25^+^ Treg are more susceptible to killing *via* Fas than CD4^+^CD25**^–^** T cells (Supporting Information Fig. 1), as shown by others [19, 20]. However, neither peritoneal CD4^+^CD25^+^ cells nor CD4^+^FoxP3^+^ cells express Fas (Supporting Information Fig. 2A), and their number is not significantly different in B16FasL-induced lavage as compared to naive lavage (Supporting Information Fig. 2B), indicating that B16FasL does not preferentially kill Treg. Finally, the data that we generated during the course of the study indicate that whilst direct Fas-FasL killing of the Treg may contribute to tumour rejection, the main contribution to tumour rejection is *via* depletion of Treg using the CD25-specific mAb PC61. Two pieces of evidence support this claim: Firstly, 100% of mice reject B16FasL after treatment with PC61 compared to approximately 50% of untreated mice. Secondly, if rejection of B16FasL was largely based on the increased susceptibility of CD4^+^CD25^+^ cells to FasL-induced cell death, the induction of a CD4^+^ T cell response, which develops as a result of Treg depletion [16], would be apparent in mice immunised with B16FasL without administration of CD25-specific depleting mAb. This CD4^+^ T cell response, which is essential for tumour rejection in 100% of the mice, is not apparent unless the antibody is administered. Thus, rejection of B16FasL is not solely based on increased susceptibility of CD4^+^CD25^+^ cells to FasL-induced cell death. As mentioned above, these data do not, however, completely exclude a role for Treg killing by the tumour, and it remains possible that expression of FasL on tumour cells may promote tumour immunity by Fas-FasL killing of Treg.

In support of our hypothesis that Treg inhibit innate immune responses, we found that adoptive transfer of CD4^+^CD25^+^ cells but not CD4^+^CD25**^–^** cells inhibited tumour rejection in RAG**^–/–^** mice. *In vivo* depletion of NK cells abolished *ex vivo* tumour lysis by peritoneal lavage cells, as did adoptive transfer of CD4^+^CD25^+^ cells, whereas depletion of Treg increased tumour killing. Together these data suggest that Treg impede the anti-tumour NK cell response. In contrast to the findings of Ghiringhelli *et al*. [21] but similar to those of Smyth *et al*. [15], we were only able to demonstrate inhibition of NK cell cytolytic activity *in vitro*after activating the purified CD4^+^CD25^+^ cells with anti-CD3 Ab and irradiated APC. Treg are believed to inhibit NKG2D-mediated NK cell killing [15], and in accordance with this observation, both tumour cell lines (B16F10 and B16FasL) used in these experiments express the NK2GD ligand Rae-1 (Fig. 2C), in contrast to other studies in which NKG2D ligands were transfected into tumour cells [14], [15]. The studies of the effect of Treg on NK cells also demonstrated that suppression by Treg could be inhibited with mAb to block TGF-β [14, 15]. In our model, TGF-β does not completely account for the suppressive effect of Treg on NK cells, since neutralisation of TGF-β using the TGF-β-specific neutralising mAb 1D11 [22] did not increase killing within the peritoneal lavage, while depletion of Treg did (data not shown). We did, however, observe that neutralisation of TGF-β increased NK cell and neutrophil recruitment into the peritoneum following injection of tumour cells. The increase, although not statistically significant, was consistent, thus raising the possibility that secretion of TGF-β, perhaps by Treg, impedes recruitment of NK cells and neutrophils. Studies of NK cell migration following virus infection support a role for MIP-1α, a chemokine whose production is driven by Type I interferons (IFN) [23, 24]. Indeed, we have previously shown that the ability to reject B16FasL is impaired in MIP-1α-deficient mice 6, a finding that may be attributable to defective NK cell migration to the tumour site. With this in mind, we are currently investigating whether Treg inhibit production of Type I IFN or MIP-1α following inoculation of mice with B16FasL cells.

Inoculation of mice with B16FasL following depletion of Treg resulted in induction of long-term tumour immunity in all injected mice. A subsequent analysis of the injected mice revealed that enhanced tumour immunity was due to the combined effects of both the cellular and the humoral arm of the immune system. As described previously, the induction of the antibody response was dependent upon the expression of FasL, whilst the induction of the CD4^+^ T cell response was dependent upon the removal of the inhibitory influence of CD25^+^ cells. We found no evidence that the quality (isotype) or quantity of the antibody (binding avidity of the serum for B16F10 and protection experiments with decreasing amount of serum) improved following depletion of Treg (data not shown) and therefore concluded that there is no direct influence of Treg on the antibody response against tumours in this model.

The observation that the combination of injection with B16FasL and depletion of CD25^+^ cells results in induction of both humoral and cellular immune responses implies that immune responses were generated against more than one, and possibly several, antigenic targets. Since tumour cells have been shown to evade immune responses by downregulating expression of antigens and/or MHC molecules, increasing the number of antigens that the immune system targets makes it more difficult for tumours to escape immune recognition (reviewed in [25]). Use of whole tumour cells, as described in this study, is therefore beneficial in this respect. It is not yet clear whether the adaptive immune responses described above are more efficiently generated in the absence of Treg due to removal of their direct inhibitory effect on T cells or due to removal of their inhibitory effect on the innate immune system, which acts as a bridge for the efficient generation of adaptive immune responses. In light of our findings that NK responses are generated more efficiently in the absence of Treg, the second possibility is intriguing. A recent report showed that activated NK cells are an important early source of IFN-γ for the priming of Th1 cells [26]. The authors of the study demonstrated that some but not all adjuvants led to the recruitment of NK cells into lymph nodes, where the cells promoted induction of Th1 responses through IFN-γ production. It is therefore possible that FasL, previously shown by ourselves and others to act as an adjuvant for DC maturation [6, 27], also acts as an adjuvant that recruits NK cells. This pathway may be amplified following Treg depletion, thereby resulting in increased production of IFN-γ. This in turn may facilitate development of the CD4^+^ T cell response, which as observed in the model described here, is capable of tumour rejection. The possibilities described above are not mutually exclusive, however, and it is likely that both pathways contribute to the overall amplification of adaptive immune responses.

In summary, we have found that immunisation of mice depleted of CD25^+^ Treg with whole tumour cells engineered to express FasL induces effective tumour immunity in all vaccinated mice. This is due to the combined effects of: 1) the pro-inflammatory properties of FasL; 2) expression of a range of antigens by whole tumour cells; and 3) removal of the inhibitory influence of CD25^+^ cells on both innate and adaptive immune responses. Such an approach, combined with the transient removal of CD25^+^ cells in patients, may significantly improve the success of vaccination following tumour resection.

## Materials and methods

### Mice and tumour cell lines

C57BL/6 (B6) and C57BL/6RAG^–/–^ (RAG^–/–^) mice (bred at Biomedical Services, Oxford or purchased from Harlan, UK) were maintained in Biomedical Services, Oxford or Cardiff. Mice aged 6–10 wks were used in all experiments. During experimental procedures mice were housed in filter-top cages in conventional facilities. The B16F10 and B16FasL cell lines used for these studies have been described previously 6. Mice were inoculated s.c. with 10^5^ or i.p. with 2 × 10^6^ tumour cells. All experiments were performed in accordance with Home Office guidelines.

### Antibodies

The hybridomas secreting anti-CD25 (PC61, rat IgG1 [28]), rat anti-*E.coli*β-galactosidase (GL113, rat IgG1, isotype control), anti-NK1.1 (PK136, mouse IgG2a 29) and anti-Gr-1 (RB6–8C5, rat IgG2b [7]) mAb as well as the efficiency of these mAb in depleting their respective cell subset have been described previously. Hybridomas were grown in culture, and mAb were purified by precipitation in saturated ammonium sulphate. For experiments involving s.c. injection of tumour cells, mice received 0.5 mg mAb PC61, GL113 and PK136 3 days and 1 day prior to tumour cell injection. Mice depleted of neutrophils received 300μg mAb RB6–8C5 1 day prior to injection of tumour cells and 1, 3, 5, and 7 days after tumour cell injection. All antibodies were injected i.p. in volumes of 100–200 μL. For depletion of macrophages, mice were injected i.p. with 1 mL autoclaved carrageenan [7] (resuspended at 2 mg/mL in PBS) 1 day prior to the injection of tumour cells and 3 and 7 days after tumour cell injection. For experiments involving i.p injection of tumour cells, mice were given 1 mg mAb 1 day prior to injection of tumour cells.

### Antibody staining and flow cytometry

Cells from mice depleted of an immune subset were stained with the following fluorescently labelled Ab: NK1.1-PE, Gr-1-PerCpCy5.5, F4/80-allophycocyanin, CD25-PE, CD4-Cy5 and FoxP3-PE. The latter required samples to be fixed and permeabilised prior to Ab staining. B16F10 and B16FasL were stained with 10 µg/mL NKG2D-Fc or GITR-L-Fc (as a control). Cells were also stained with anti-Rae-1 (goat IgG), anti-MHC class I (mouse IgG1) or appropriate isotype controls.

### Purification of CD4^+^CD25^+^ T cells

CD4^+^CD25^+^ T cells were purified by negative selection using Dynabeads and subsequent positive selection using MACS beads. Spleen and lymph node cell suspensions prepared from naive B6 mice were resuspended at 10^8^ cells/mL in HBSS (Gibco) and mixed at a 2:1 vol:vol ratio with an Ab cocktail containing 10 μg/mL rat anti-B220, anti-Mac-1, anti-CD8, anti-MHC class II and anti-NK1.1 in HBSS/0.1% BSA in order to enrich CD4^+^ cells. After a 20 min incubation on ice, the cells were washed twice in HBSS, and Dynabead-conjugated sheep anti-rat IgG Ab (Dynal) was added at a ratio of 1 Dynabead per spleen cell. After a further 20 min incubation at 4°C, Dynabead-bound cells were magnetically separated according to the manufacturer's instructions (Dynal). Dynabead-bound cells were discarded, a quarter of the original number of Dynabeads was added to the cells, and a second round of negative selection was performed as above. Cells that were not Dynabead-bound (enriched for CD4^+^ cells) were subsequently incubated with anti-CD25 Ab conjugated to PE (Miltenyi) and purified using microbeads conjugated to anti-PE Ab according to the manufacturer's instructions (Miltenyi). Alternatively, CD4^+^CD25^+^ and CD4^+^CD25^–^ cells were purified using the Miltenyi purification kit. A purity of greater than 90% CD4^+^CD25^+^ cells was obtained in all experiments.

### Peritoneal model and killing assay

Mice were injected i.p. with 2 × 10^6^ B16F10 or B16FasL cells. The peritoneal lavage was collected 18 h later using PBS with 2 mM EDTA and 0.5% BSA. Cells were then either subjected to FACS analysis or used as effectors in a ^51^Cr-release assay. Briefly, targets were labelled with ^51^Cr, and effectors were added at the indicated ratio for 4 h before the supernatant was collected. Percent tumour lysis was calculated using the following formula: [sample – background (no effectors)] / [total lysis – background].

### CD25^+^ Treg suppression assay

CD4^+^CD25^+^ Treg from spleens and lymph nodes were purified using the Miltenyi purification kit and stimulated for 18 h with 1 µm anti-CD3 and 10^5^ irradiated CD4^–^ splenocytes. B16FasL cells (2 × 10^6^) were injected into B6 mice, and 24 h later the peritoneum was lavaged and the cells counted. Lavaged cells (10^5^) were added to 10^4^ B16F10 cells previously labelled with ^51^Cr in the presence of 10^5^ purified CD4^+^CD25^+^ or CD4^+^CD25^–^ T cells. Controls included lavaged cells (effectors) alone and CD4^+^CD25^–^ cells without effectors. The supernatant was collected 5 h later and analysed on a beta counter. Means were calculated from triplicates. To test whether CD25^+^ Treg were functional, a proliferation assay was performed in the presence of Treg. Purified CD4^+^CD25^+^ were cultured in the presence of 10^5^ CD4^+^CD25^–^ splenocytes, 1 µm anti-CD3 and 10^5^ irradiated CD4^–^ splenocytes. Cells were incubated for 3 days and pulsed with [^3^H]-thymidine for a further 18 h. Means were calculated from triplicates. At suppressor/effector ratios of 1:100, 1:10, 1:1 and 10:1, 10%, 38%, 59% and 82% inhibition was observed, respectively.

### Transfer of CD4^+^ cells and serum

CD4^+^ lymphocytes from spleens were purified by positive selection using magnetic beads (Miltenyi Biotec) according to the manufacturer's instructions. Purity of CD4^+^ cells was greater than 95%. Recipient mice were injected i.v. with 5 × 10^6^ cells on the day of purification. To obtain serum, blood was clotted at 37°C for 1 h, kept at 4°C for at least 1 h and then spun at 20 000 × g for 10 min. Recipient mice received the serum i.v. (200 µL). In all transfer experiments, mice were inoculated the following day with tumour cells.

## Abbreviations

B6: C57BL/6
RAG^–/–^: C51BL/6RAG^–/–^
