# Supplementary material for: Regulatory T cells inhibit Fas ligand-induced innate and adaptive tumour immunity
Source: Eur J Immunol. 2007 Mar;37(3):758–67. doi: 10.1002/eji.200636593 (PMC2435420; doi:10.1002/eji.200636593)
Supplement: Supplementary file 1 [file eji0037-0758-SD1.pdf]

# European Journal of Immunology

## Supporting Information

## for

## Eur. J. Immunol. 10.1002/eji.200736593

**Regulatory T cells inhibit Fas ligand-induced innate and adaptive tumour immunity**

Anna-Katharina Simon, Emma Jones, Hannah Richards, Kate Wright, Gareth Betts, Andrew Godkin, Gavin Screaton and Awen Gallimore

© 2007 WILEY-VCH Verlag GmbH & Co. KGaA, Weinheim **www.eji-journal.eu**

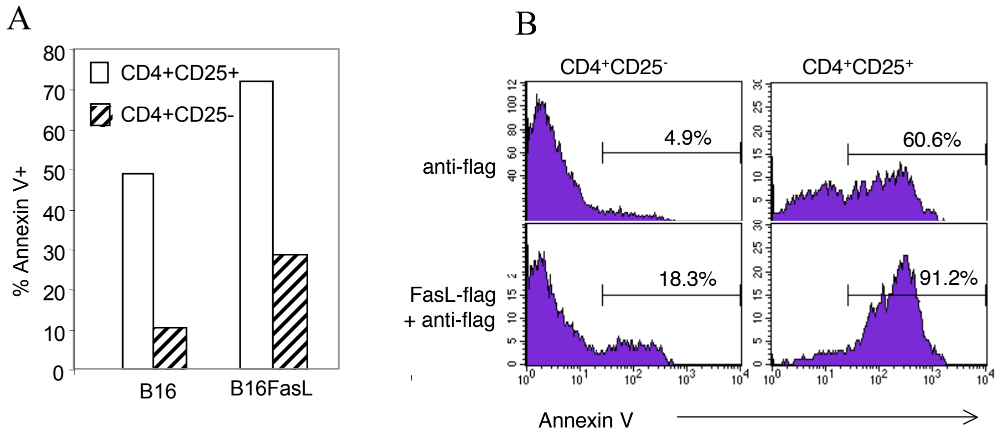


**Supplementary Figure 1** CD4$^{+}$CD25$^{+}$ are more susecptible to apoptosis than CD4$^{+}$CD25$^{-}$

**A** Splenocytes were cultured in the presence of B16FasL or B16 at 1:10 ratio for 6 hours. Splenocytes were stained with antibodies against CD4 and CD25 followed by Annexin V-FITC at RT in Annexin V staining buffer. For the FACS analysis gates were first set on live gates, and then gated on CD4$^{+}$CD25$^{+}$ or CD4$^{+}$CD25$^{-}$ cells. This is one representative experiment of two performed **B** Splenocytes were cultured for 18h with FasL-flag with or without anti-flag (5µg/ml M2 Sigma). Staining was performed as in A. This experiment was repeated twice.

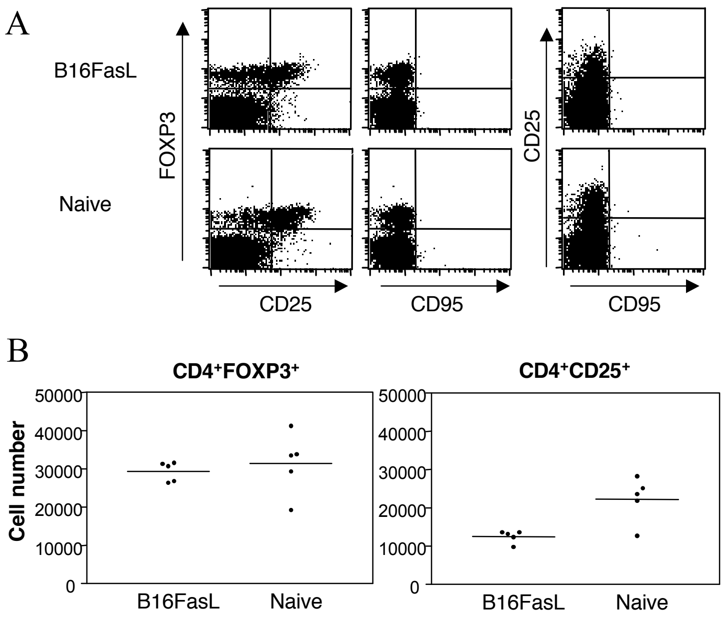


**Supplementary Figure 2** Peritoneal lavage contains FoxP3$^{+}$ cells which are negative for Fas

**A** B6 mice were either inoculated intraperitoneally with B16FasL or remained untreated. 18 hours later peritoneal cells were recovered by lavage and immunostained for CD4, FoxP3, CD25 and CD95 (Fas). Samples were then evaluated by FACS and plots shown are representative of 5 mice per group. Total numbers of CD4$^{+}$FoxP3$^{+}$ and CD4$^{+}$CD25$^{+}$ cells in the lavage are also given in **B** and **C**.
